# Supplementary material for: Feature selection for the accurate prediction of septic and cardiogenic shock ICU mortality in the acute phase
Source: PLoS One. 2018 Nov 20;13(11):e0199089. doi: 10.1371/journal.pone.0199089 (PMC6245679; doi:10.1371/journal.pone.0199089)
Supplement: S1 Appendix — The resulting features from applying multiple FS techniques to the three available datasets, their stability scores, additional CBNs and a full list of ShockOmics attributes. (PDF) [file pone.0199089.s001.pdf]

S1 Appendix:
Complementary material for the feature selection and causal discovery experiments

Contents

1 The T1 dataset **4**
- 1.1 Univariate feature selection . . . 4
- 1.2 Recursive feature elimination . . . 6
- 1.3 UFS + RFE . . . 8
- 1.4 Random Forest . . . 10
- 1.5 Aggregated features . . . 12

2 The T1+T2 dataset **15**
- 2.1 Univariate feature selection . . . 15
- 2.2 Recursive feature elimination . . . 17
- 2.3 UFS + RFE . . . 19
- 2.4 Random Forest . . . 21
- 2.5 Aggregated features . . . 23

3 The Full dataset **26**
- 3.1 Univariate feature selection . . . 26
- 3.2 Recursive feature elimination . . . 28
- 3.3 UFS + RFE . . . 30
- 3.4 Random Forest . . . 32
- 3.5 Aggregated features . . . 34

4 Additional causal Bayesian networks **36**

5 Full list of ShockOmics attributes **41**

List of Tables

1 **T1** dataset selected features and their stability scores for the *UFS* feature selection method. . . . 5

2 **T1** dataset selected features and their stability scores for the *RFE* feature selection method. . . . 7

3 **T1** dataset selected features and their stability scores for the *UFS+RFE* feature selection method (the UFS preselected 80 features and the RFE chose the rest). . . . 9

4 **T1** dataset selected features and their stability scores for the *Random Forest* feature selection method. . . . 11

5 **T1** dataset aggregated selected features and their stability scores for all feature selection methods. . . . 13

6 **T1+T2** dataset selected features and their stability scores for the *UFS* feature selection method. . . . 16

7 **T1+T2** dataset selected features and their stability scores for the *RFE* feature selection method. . . . 18

8 **T1+T2** dataset selected features and their stability scores for the *UFS+RFE* feature selection method (the UFS preselected 120 features and the RFE chose the rest). . . . 20

9 **T1+T2** dataset selected features and their stability scores for the *Random Forest* feature selection method. . . . 22
10 **T1+T2** dataset aggregated selected features and their stability scores for all feature selection methods. . . . 24
11 **Full** dataset selected features and their stability scores for the *UFS* feature selection. 27
12 **Full** dataset selected features and their stability scores for the *RFE* feature selection method. . . . 29
13 **Full** dataset selected features and their stability scores for the *UFS+RFE* feature selection method (the UFS preselected 120 features and the RFE chose the rest). . 31
14 **Full** dataset selected features and their stability scores for the *Random Forest* feature selection method. . . . 33
15 **Full** dataset aggregated selected features and their stability scores for all feature selection methods. . . . 35
16 Full list of the ShockOmics dataset attributes. . . . 47

List of Figures

1 **The *(T1, Aggr.) CBN*.** The CBN for the *(T1, Aggr.)* feature set obtained: a) without the target feature; b) with the target feature. . . . 36
2 **The *(T1+T2, Aggr.)* CBN.** The CBN for the *(T1+T2, Aggr.)* feature set obtained: a) without the target feature; b) with the target feature. . . . 37
3 **The *(Full, RF)* CBN.** The CBN for the *(Full, RF)* feature set obtained: a) without the target feature; b) with the target feature. . . . 38
4 **The *(Full, Aggr.)* CBN.** The CBN for the *(Full, Aggr.)* feature set obtained: a) without the target feature; b) with the target feature. . . . 39

1 The T1 dataset

1.1 Univariate feature selection

Rank	Feature name	Score
1	APACHE II_T1	0.98
2	SOFA_T1	0.86
3	LA dilatation by eyeballing_T1	0.7
4	Respiratory rate_T1	0.53
5	* Norepinephrine (μg/kg/min)_T1	0.46
6	Tidal volume (VT)_T1	0.4
7	Number of affected organs	0.39
8	Glasgow Coma Scale_T1	0.39
9	Pulmonary artery systolic pressure (TR jet by CW + CVP) (mmHg)_T1	0.36
10	Fluid Balance (ml)_T1	0.36
11	Platelet count_T1	0.36
12	Base Excess (mmol/L)_T1	0.35
13	Tricuspid regurgitation maximal velocity (by CW) (cm/s)_T1	0.35
14	Urine Output (mL/day)_T1	0.35
15	LV Dilatation (LVEDV)_T1=Moderate	0.29
16	PCT_Value (mg/mL)_T1	0.27
17	Aortic Valve Stenosis_T1=Moderate	0.27
18	LVOT average diameter (mm)_T1	0.26
19	Hypotension (SBP $<$ 90 mmHg or MAP $<$ 70 mmHg or SBP decrease $>$ 40 mmHg)_T1	0.24
20	Anuria and/or RRT_T1	0.24
21	Mode_T1=Volume controlled	0.24
22	K Ur_T1	0.23
23	Site of sampling=Urine	0.23
24	Is the patient under other drugs_T1	0.23
25	Lactate levels (mmol/L)_T1	0.22
26	Sedation Scale (SAS)_T1	0.2
27	Pplat_T1	0.17
28	Respiratory rate (rpm)_T1	0.16
29	Site of sampling=CSF	0.15
30	FiO2_T1	0.14
31	Temperature (°C)_T1	0.14
32	Site of sampling.1=Abdominal drain	0.14
33	Mode_T1=Pressure controlled	0.14
34	Site of sampling.2=Urine	0.14
35	* Dobutamine (μg/kg/min)_T1	0.13
36	HCO3 (mmol/L)_T1	0.12
37	Sat O2/FiO2_T1	0.12
38	PEEP_T1	0.11
39	PaCO2 (mmHg)_T1	0.11
40	pH_T1	0.1
41	E wave (cm/s)_T1	0.1
42	Platelets (10^3/mm^3)_T1	0.09
43	RBC count_T1	0.08
44	Lympho abs count_T1	0.08
45	Result=Positive	0.08
46	SvcO2 (%)_T1	0.08
47	Is the patient under sedation drugs_T1	0.07
48	Result=Negative	0.07
49	LV Hypertrophy (LV mass)_T1=Moderate	0.07
50	Acute Kidney Injury_T1=AKIN III	0.06

51	Aortic Valve Regurgitation_T1=Severe	0.06
52	Value (%)_T1	0.06
53	Is the patient under inotropic drugs_T1	0.06
54	Tricuspid Valve Regurgitation_T1=Mild	0.05
55	The patient was already receiving antibiotic treatment and this coverage has been maintained until the onset of sepsis	0.05
56	Tricuspid Valve Regurgitation_T1=Moderate	0.05
57	Mitral Valve Regurgitation_T1=No valvular assessment available	0.05
58	Site of sampling=Other	0.05
59	Tricuspid annular tissular doppler S wave (DTI) (cm/s)_T1	0.04
60	Mean arterial pressure (mmHg)_T1	0.04
61	Diastolic Blood Pressure (mmHg)_T1	0.04
62	Aortic Valve Regurgitation_T1=Moderate	0.04
63	Is the patient affected by Prolonged Arrhythmias (PA)_T1	0.03
64	Rhythm_T1=Atrial fibrillation	0.03
65	Troponine_T1	0.03
66	Aortic Valve Stenosis_T1=No valvular dysfunction	0.02
67	Rhythm_T1=Sinus	0.02
68	Site of sampling=Respiratory tract	0.02
69	PaO2/FiO2_T1	0.02
70	Neutro abs count_T1	0.02
71	Na (mmol/L)_T1	0.02
72	Site of sampling.1=Respiratory tract	0.02
73	Aortic Valve Regurgitation_T1=No valvular dysfunction	0.02
74	E/e'_T1	0.02
75	Glycemia (mg/dL)_T1	0.02
76	Creatinine (mg/dL)_T1	0.02
77	Was the patient transfused_T1	0.02
78	Systolic blood pressure (mmHg)_T1	0.01
79	Was shock diagnosed within 48 from hospital admission	0.01
80	Mitral Valve Regurgitation_T1=No valvular dysfunction	0.01

Table 1: **T1** dataset selected features and their stability scores for the *UFS* feature selection method.

1.2 Recursive feature elimination

Rank	Feature name	Score
1	Glasgow Coma Scale_T1	0.75
2	K Ur_T1	0.67
3	Platelet count_T1	0.6
4	Tricuspid regurgitation maximal velocity (by CW) (cm/s)_T1	0.58
5	Respiratory rate_T1	0.57
6	APACHE II_T1	0.57
7	PT_T1	0.57
8	HCO3 (mmol/L)_T1	0.54
9	Hematocrit (%)_T1	0.54
10	Tidal volume (VT)_T1	0.48
11	Heart rate (bpm)_T1.1	0.48
12	Inferior vena cava distensibility index (%)_T1	0.46
13	Value (%)_T1	0.44
14	Tricuspid annular tissular doppler S wave (DTI) (cm/s)_T1	0.43
15	Na Ur_T1	0.39
16	aPTT_T1	0.37
17	SOFA_T1	0.36
18	Creat Ur_T1	0.33
19	E wave (cm/s)_T1	0.32
20	A wave (cm/s)_T1	0.31
21	Respiratory rate (rpm)_T1	0.31
22	Systolic blood pressure (mmHg)_T1	0.29
23	* Dobutamine (μg/kg/min)_T1	0.29
24	Leukocytes (total*1000/mm^3)_T1	0.28
25	Na (mmol/L)_T1	0.27
26	Base Excess (mmol/L)_T1	0.26
27	PaO2 (mmHg)_T1	0.25
28	Weight (kg)	0.25
29	PCT_Value (mg/mL)_T1	0.24
30	Tricuspid annular plane systolic excursion (TAPSE) (mm)_T1	0.24
31	SvcO2 (%)_T1	0.23
32	Neutro abs count_T1	0.22
33	E wave deceleration time (ms)_T1	0.22
34	Height (cm)	0.22
35	Pulmonary artery systolic pressure (TR jet by CW + CVP) (mmHg)_T1	0.21
36	Sat O2/FiO2_T1	0.21
37	PaO2/FiO2_T1	0.2
38	WBC abs count_T1	0.19
49	Diastolic Blood Pressure (mmHg)_T1	0.18
40	Bilirubin (mg/dL)_T1	0.18
41	Pplat_T1	0.17
42	PaCO2 (mmHg)_T1	0.16
43	Sat O2 (%)_T1	0.16
44	Heart rate (bpm)_T1	0.15
45	Lympho abs count_T1	0.14
46	Chloride_T1	0.14
47	Fibrinogen_T1 (g/L)	0.11
48	BMI	0.11
49	LVOT average velocity-time integral (cm)_T1	0.11
50	Creatinine (mg/dL)_T1	0.11

51	LVOT average diameter (mm)_T1	0.1
52	E/A ratio_T1	0.08
53	Mean arterial pressure (mmHg)_T1	0.07
54	E/e'_T1	0.06
55	Lateral e' (cm/s)_T1	0.05
56	Number of affected organs	0.05
57	Lactate levels (mmol/L)_T1	0.04
58	CRP_Value (mg/L)_T1	0.04
59	PEEP_T1	0.04
60	Platelets ($10^3/mm^3$)_T1	0.03
61	Glycemia (mg/dL)_T1	0.02
62	LA dilatation by eyeballing_T1	0.02
63	Sedation Scale (SAS)_T1	0.01
64	Temperature (°C)_T1	0.01
65	Result=Positive	0.01
66	Result=Negative	0.01
67	Mitral Valve Regurgitation_T1=Mild	0.01
68	Is the patient under inotropic drugs_T1	0.01

Table 2: **T1** dataset selected features and their stability scores for the *RFE* feature selection method.

1.3 UFS + RFE

Rank	Feature name	Score
1	Glasgow Coma Scale_T1	0.69
2	SOFA_T1	0.6
3	HCO3 (mmol/L)_T1	0.56
4	Respiratory rate_T1	0.44
5	APACHE II_T1	0.42
6	K Ur_T1	0.34
7	Respiratory rate (rpm)_T1	0.3
8	Pplat_T1	0.3
9	Aortic Valve Regurgitation_T1=No valvular dysfunction	0.29
10	Base Excess (mmol/L)_T1	0.28
11	Lympho abs count_T1	0.28
12	LA dilatation by eyeballing_T1	0.28
13	Tricuspid regurgitation maximal velocity (by CW) (cm/s)_T1	0.27
14	Tricuspid annular tissular doppler S wave (DTI) (cm/s)_T1	0.26
15	Neutro abs count_T1	0.26
16	Platelet count_T1	0.26
17	Fibrinogen_T1 (g/L)	0.26
18	Lactate levels (mmol/L)_T1	0.25
19	Sample 1=Negative	0.24
20	Sample 1=Positive	0.24
21	Sedation Scale (SAS)_T1	0.23
22	Number of affected organs	0.23
23	Inferior vena cava distensibility index (%)_T1	0.23
24	Value (%)_T1	0.23
25	* Dobutamine (μg/kg/min)_T1	0.23
26	LVOT average diameter (mm)_T1	0.22
27	Site of sampling=Urine	0.21
28	Tidal volume (VT)_T1	0.19
29	Temperature (°C)_T1	0.19
30	SvcO2 (%)_T1	0.19
31	Pulmonary artery systolic pressure (TR jet by CW + CVP) (mmHg)_T1	0.18
32	Diastolic Blood Pressure (mmHg)_T1	0.18
33	PCT_Value (mg/mL)_T1	0.17
34	Site of sampling=CSF	0.17
35	Na Ur_T1	0.17
36	E/e'_T1	0.17
37	PEEP_T1	0.16
38	PT_T1	0.16
39	Tricuspid annular plane systolic excursion (TAPSE) (mm)_T1	0.15
40	E wave (cm/s)_T1	0.15
41	Weight (kg)	0.15
42	Bilirubin (mg/dL)_T1	0.14
43	BMI	0.14
44	Systolic blood pressure (mmHg)_T1	0.13
45	PaCO2 (mmHg)_T1	0.13
46	Heart rate (bpm)_T1	0.12
47	Mean arterial pressure (mmHg)_T1	0.12
48	RBC count_T1	0.1
49	Sat O2 (%)_T1	0.09
50	LVOT average velocity-time integral (cm)_T1	0.09

51	Is the patient under other drugs_T1	0.09
52	Midazolam (μg/kg/min)_T1	0.09
53	Creatinine (mg/dL)_T1	0.08
54	Mode_T1=Volume controlled	0.08
55	* Norepinephrine (μg/kg/min)_T1	0.08
56	Is the patient under inotropic drugs_T1	0.08
57	Acute Kidney Injury_T1=AKIN III	0.07
58	Tricuspid Valve Regurgitation_T1=Mild	0.07
59	Lateral e' (cm/s)_T1	0.07
60	Result=Negative	0.07
61	Sat O2/FiO2_T1	0.06
62	Mitral Valve Regurgitation_T1=No valvular dysfunction	0.06
63	PaO2/FiO2_T1	0.06
64	Result=Positive	0.06
65	Na (mmol/L)_T1	0.06
66	A wave (cm/s)_T1	0.06
67	E wave deceleration time (ms)_T1	0.06
68	LV Hypertrophy (LV mass)_T1=Moderate	0.06
69	aPTT_T1	0.05
70	Mitral Valve Regurgitation_T1=No valvular assessment available	0.05
71	Is the patient affected by Prolonged Arrhythmias (PA)_T1	0.05
72	Blood Cultures	0.05
73	Creat Ur_T1	0.05
74	Aortic Valve Regurgitation_T1=Moderate	0.05
75	K (mmol/L)_T1	0.05
76	Blood Cultures=Not performed	0.05
77	Was shock diagnosed within 48 from hospital admission	0.04
78	Anuria and/or RRT_T1	0.04
79	Site of sampling=Respiratory tract	0.04
80	PaO2 (mmHg)_T1	0.04

Table 3: **T1** dataset selected features and their stability scores for the *UFS+RFE* feature selection method (the UFS preselected 80 features and the RFE chose the rest).

1.4 Random Forest

Rank	Feature name	Score
1	APACHE II_T1	0.72
2	SOFA_T1	0.56
3	Respiratory rate_T1	0.54
4	Urine Output (mL/day)_T1	0.52
5	E wave (cm/s)_T1	0.48
6	Fluid Balance (ml)_T1	0.46
7	K Ur_T1	0.44
8	Lactate levels (mmol/L)_T1	0.39
9	Tidal volume (VT)_T1	0.39
10	Pulmonary artery systolic pressure (TR jet by CW + CVP) (mmHg)_T1	0.38
11	* Norepinephrine (μg/kg/min)_T1	0.37
12	PCT_Value (mg/mL)_T1	0.35
13	Heart rate (bpm)_T1	0.33
14	E/e'_T1	0.33
15	PEEP_T1	0.31
16	Pplat_T1	0.3
17	Platelet count_T1	0.29
18	Na Ur_T1	0.29
19	pH_T1	0.27
20	PaO2/FiO2_T1	0.26
21	LA dilatation by eyeballing_T1	0.26
22	Fibrinogen_T1 (g/L)	0.26
23	Glycemia (mg/dL)_T1	0.26
24	* Dobutamine (μg/kg/min)_T1	0.26
25	Tricuspid regurgitation maximal velocity (by CW) (cm/s)_T1	0.25
26	LVOT average velocity-time integral (cm)_T1	0.24
27	Temperature (°C)_T1	0.23
28	LVOT average diameter (mm)_T1	0.23
29	Creat Ur_T1	0.22
30	Platelets (10^3/mm^3)_T1	0.21
31	A wave (cm/s)_T1	0.21
32	Troponine_T1	0.21
33	Tricuspid annular tissular doppler S wave (DTI) (cm/s)_T1	0.2
34	Neutro abs count_T1	0.2
35	Heart rate (bpm)_T1.1	0.2
36	Lympho abs count_T1	0.19
37	SvcO2 (%)_T1	0.19
38	Glasgow Coma Scale_T1	0.19
39	Tricuspid annular plane systolic excursion (TAPSE) (mm)_T1	0.18
40	Base Excess (mmol/L)_T1	0.18
41	Respiratory rate (rpm)_T1	0.18
42	E wave deceleration time (ms)_T1	0.18
43	PaCO2 (mmHg)_T1	0.17
44	Midazolam (μg/kg/min)_T1	0.17
45	Mean arterial pressure (mmHg)_T1	0.16
46	Diastolic Blood Pressure (mmHg)_T1	0.16
47	Number of affected organs	0.16
48	aPTT_T1	0.15
49	Inferior vena cava distensibility index (%)_T1	0.15
50	Na (mmol/L)_T1	0.15

51	Sat O2/FiO2_T1	0.14
52	RBC count_T1	0.14
53	Leukocytes (total*1000/mm^3)_T1	0.14
54	E/A ratio_T1	0.13
55	Hematocrit (%)_T1	0.13
56	K (mmol/L)_T1	0.13
57	HCO3 (mmol/L)_T1	0.12
58	WBC abs count_T1	0.12
59	BMI	0.12
60	Sedation Scale (SAS)_T1	0.11
61	Bilirubin (mg/dL)_T1	0.11
62	PT_T1	0.11
63	Lateral e' (cm/s)_T1	0.11
64	Weight (kg)	0.11
65	CRP_Value (mg/L)_T1	0.1
66	Cardiac output (ml/min)_T1	0.09
67	Creatinine (mg/dL)_T1	0.09
68	Prothrombin time (INR)_T1	0.08
69	PaO2 (mmHg)_T1	0.08
70	Sample 1=Positive	0.08
71	Value (%)_T1	0.08
72	Is the patient under other drugs_T1	0.08
73	Systolic blood pressure (mmHg)_T1	0.07
74	FiO2_T1	0.07
75	Aortic Valve Regurgitation_T1=No valvular dysfunction	0.07
76	Chloride_T1	0.07
77	Sat O2 (%)_T1	0.06
78	Tricuspid Valve Regurgitation_T1=Moderate	0.06
79	Mitral Valve Regurgitation_T1=No valvular dysfunction	0.05
80	Mitral Valve Regurgitation_T1=No valvular assessment available	0.05

Table 4: **T1** dataset selected features and their stability scores for the *Random Forest* feature selection method.

1.5 Aggregated features

Rank	Feature name	Score
1	APACHE II_T1	0.6725
2	SOFA_T1	0.595
3	Respiratory rate_T1	0.52
4	Glasgow Coma Scale_T1	0.505
5	K Ur_T1	0.42
6	Platelet count_T1	0.3775
7	Tidal volume (VT)_T1	0.365
8	Tricuspid regurgitation maximal velocity (by CW) (cm/s)_T1	0.3625
9	HCO3 (mmol/L)_T1	0.335
10	LA dilatation by eyeballing_T1	0.315
11	Pulmonary artery systolic pressure (TR jet by CW + CVP) (mmHg)_T1	0.2825
12	Base Excess (mmol/L)_T1	0.2675
13	E wave (cm/s)_T1	0.2625
14	PCT_Value (mg/mL)_T1	0.2575
15	Respiratory rate (rpm)_T1	0.2375
16	Pplat_T1	0.235
17	Tricuspid annular tissular doppler S wave (DTI) (cm/s)_T1	0.2325
18	* Norepinephrine (μg/kg/min)_T1	0.2275
19	* Dobutamine (μg/kg/min)_T1	0.2275
20	Lactate levels (mmol/L)_T1	0.225
21	Urine Output (mL/day)_T1	0.2175
22	Inferior vena cava distensibility index (%)_T1	0.2125
23	Na Ur_T1	0.2125
24	PT_T1	0.21
25	Number of affected organs	0.2075
26	Fluid Balance (ml)_T1	0.205
27	Value (%)_T1	0.2025
28	LVOT average diameter (mm)_T1	0.2025
29	Neutro abs count_T1	0.175
30	Heart rate (bpm)_T1.1	0.175
31	Lympho abs count_T1	0.1725
32	SvcO2 (%)_T1	0.1725
33	Hematocrit (%)_T1	0.1725
34	Fibrinogen_T1 (g/L)	0.16
35	PEEP_T1	0.155
36	Heart rate (bpm)_T1	0.15
37	Creat Ur_T1	0.15
38	E/e'_T1	0.145
39	A wave (cm/s)_T1	0.145
40	Tricuspid annular plane systolic excursion (TAPSE) (mm)_T1	0.1425
41	Temperature (°C)_T1	0.1425
42	aPTT_T1	0.1425
43	PaCO2 (mmHg)_T1	0.1425
44	Diastolic Blood Pressure (mmHg)_T1	0.14
45	Sedation Scale (SAS)_T1	0.1375
46	PaO2/FiO2_T1	0.135
47	Sat O2/FiO2_T1	0.1325
48	Weight (kg)	0.1275
49	Systolic blood pressure (mmHg)_T1	0.125
50	Na (mmol/L)_T1	0.125

51	E wave deceleration time (ms)_T1	0.115
52	LVOT average velocity-time integral (cm)_T1	0.1125
53	Leukocytes (total*1000/mm^3)_T1	0.1125
54	Site of sampling=Urine	0.11
55	Bilirubin (mg/dL)_T1	0.1075
56	Is the patient under other drugs_T1	0.1
57	Mean arterial pressure (mmHg)_T1	0.0975
58	Aortic Valve Regurgitation_T1=No valvular dysfunction	0.095
59	pH_T1	0.0925
60	Platelets (10^3/mm^3)_T1	0.0925
61	BMI	0.0925
62	Site of sampling=CSF	0.0925
63	PaO2 (mmHg)_T1	0.0925
64	Mode_T1=Volume controlled	0.09
65	LV Dilatation (LVEDV)_T1=Moderate	0.0825
66	RBC count_T1	0.08
67	Anuria and/or RRT_T1	0.08
68	Sample 1=Positive	0.08
69	Aortic Valve Stenosis_T1=Moderate	0.08
70	Sat O2 (%)_T1	0.0775
71	WBC abs count_T1	0.0775
72	Glycemia (mg/dL)_T1	0.075
73	Creatinine (mg/dL)_T1	0.075
74	Sample 1=Negative	0.07
75	Hypotension (SBP $<$ 90 mmHg or MAP $<$ 70 mmHg or SBP decrease $>$ 40 mmHg)_T1	0.0675
76	Height (cm)	0.065
77	Midazolam (μg/kg/min)_T1	0.065
78	Chloride_T1	0.06
79	E/A ratio_T1	0.06
80	Lateral e' (cm/s)_T1	0.0575

Table 5: **T1** dataset aggregated selected features and their stability scores for all feature selection methods.

2 The T1+T2 dataset

2.1 Univariate feature selection

Rank	Feature name	Score
1	SOFA_T2	1.0
2	APACHE II_T1	0.9
3	APACHE II_T2	0.62
4	LA dilatation by eyeballing_T1	0.45
5	SOFA_T1	0.4
6	Lactate levels (mmol/L)_T2	0.34
7	Glasgow Coma Scale_T2	0.31
8	* Norepinephrine (μg/kg/min)_T2	0.28
9	pH_T2	0.27
10	FiO2_T2	0.25
11	* Norepinephrine (μg/kg/min)_T1	0.24
12	Sedation Scale (SAS)_T2	0.21
13	Respiratory rate_T1	0.21
14	Tricuspid regurgitation maximal velocity (by CW) (cm/s)_T1	0.21
15	Pulmonary artery systolic pressure (TR jet by CW + CVP) (mmHg)_T1	0.2
16	Tracheal Intubation_T2	0.17
17	Platelet count_T1	0.17
18	Tidal volume (VT)_T2	0.16
19	Creatinine (mg/dL)_T2	0.16
20	Hypotension (SBP $<$ 90 mmHg or MAP $<$ 70 mmHg or SBP decrease $>$ 40 mmHg)_T2	0.15
21	Platelet count_T2	0.15
22	Sat O2/FiO2_T2	0.14
23	Number of affected organs	0.14
24	Tidal volume (VT)_T1	0.13
25	K (mmol/L)_T2	0.13
26	Fluid Balance (ml)_T1	0.11
27	PT_T2	0.11
28	Glasgow Coma Scale_T1	0.11
29	Prothrombin time (INR)_T2	0.1
30	Acute Kidney Injury_T2=AKIN III	0.1
31	Neutro abs count_T2	0.1
32	LV Dilatation (LVEDV)_T1=Moderate	0.1
33	Urine Output (mL/day)_T1	0.1
34	RBC count_T2	0.09
35	Urine Output (mL/day)_T2	0.09
36	Troponine_T2	0.09
37	LVOT average diameter (mm)_T1	0.09
38	PCT_Value (mg/mL)_T1	0.08
39	Base Excess (mmol/L)_T1	0.08
40	Tricuspid Valve Regurgitation_T2=Moderate	0.08
41	Anuria and/or RRT_T1	0.08
42	Is the patient under sedation drugs_T2	0.08
43	Is the patient under other drugs_T1	0.08
44	K Ur_T1	0.07
45	Aortic Valve Stenosis_T1=Moderate	0.07
46	Mode_T1=Volume controlled	0.06
47	Site of sampling=CSF	0.05
48	Fluid Balance (ml)_T2	0.05
49	SvcO2 (%)_T2	0.05
50	Tricuspid Valve Regurgitation_T2=Mild	0.05

51	HCO3 (mmol/L)_T2	0.04
52	Platelets (10^3/mm^3)_T2	0.04
53	Respiratory rate (rpm)_T1	0.04
54	SvcO2 (%)_T1	0.04
55	Site of sampling=Urine	0.04
56	Tricuspid Valve Regurgitation_T2=No valvular dysfunction	0.04
57	Site of sampling.1=Abdominal drain	0.04
58	Pplat_T1	0.04
59	* Dobutamine (μg/kg/min)_T1	0.04
60	Site of sampling.2=Urine	0.04
61	Sedation Scale (SAS)_T1	0.03
62	Lactate levels (mmol/L)_T1	0.03
63	Hypotension (SBP $<$ 90 mmHg or MAP $<$ 70 mmHg or SBP decrease $>$ 40 mmHg)_T1	0.03
64	Anuria and/or RRT_T2	0.03
65	Temperature (°C)_T1	0.03
66	Respiratory rate_T2	0.03
67	Na Ur_T2	0.03
68	Mode_T2=Volume controlled	0.03
69	Creat Ur_T2	0.03
70	A wave (cm/s)_T2	0.03
71	Mode_T2=Pressure controlled	0.03
72	HCO3 (mmol/L)_T1	0.02
73	Acute Kidney Injury_T1=AKIN III	0.02
74	Tricuspid annular tissular doppler S wave (DTI) (cm/s)_T1	0.02
75	Base Excess (mmol/L)_T2	0.02
76	Segments affected by eyeballing_T2	0.02
77	Pplat_T2	0.02
78	PaO2/FiO2_T2	0.02
79	RBC count_T1	0.02
80	Lympho abs count_T1	0.02

Table 6: **T1+T2** dataset selected features and their stability scores for the *UFS* feature selection method.

2.2 Recursive feature elimination

Rank	Feature name	Score
1	APACHE II_T1	0.59
2	Tricuspid regurgitation maximal velocity (by CW) (cm/s)_T1	0.56
3	Platelet count_T1	0.55
4	Leukocytes (total*1000/mm^3)_T2	0.55
5	Neutro abs count_T2	0.48
6	E wave (cm/s)_T1	0.48
7	Glasgow Coma Scale_T1	0.46
8	Heart rate (bpm)_T1.1	0.46
9	Tidal volume (VT)_T1	0.43
10	K Ur_T1	0.42
11	PT_T1	0.41
12	Hematocrit (%)_T1	0.41
13	HCO3 (mmol/L)_T1	0.4
14	Respiratory rate (rpm)_T1	0.38
15	E wave deceleration time (ms)_T1	0.32
16	Na (mmol/L)_T1	0.31
17	PaO2 (mmHg)_T1	0.3
18	Na Ur_T1	0.3
19	PaO2 (mmHg)_T2	0.28
20	WBC abs count_T2	0.27
21	Respiratory rate_T2	0.27
22	Glasgow Coma Scale_T2	0.27
23	Value (%)_T1	0.27
24	Sat O2/FiO2_T1	0.26
25	Inferior vena cava distensibility index (%)_T1	0.26
26	A wave (cm/s)_T2	0.26
27	Weight (kg)	0.26
28	Base Excess (mmol/L)_T1	0.25
29	K Ur_T2	0.25
30	aPTT_T2	0.24
31	PaO2/FiO2_T1	0.24
32	Hematocrit (%)_T2	0.24
33	Na Ur_T2	0.24
34	PT_T2	0.23
35	CRP_Value (mg/L)_T2	0.22
36	Platelets (10^3/mm^3)_T2	0.21
37	Creat Ur_T1	0.2
38	CRP_Value (mg/L)_T1	0.19
39	PaCO2 (mmHg)_T1	0.18
40	E wave deceleration time (ms)_T2	0.18
41	PCT_Value (mg/mL)_T1	0.17
42	Respiratory rate_T1	0.17
43	Tricuspid annular tissular doppler S wave (DTI) (cm/s)_T1	0.16
44	Systolic blood pressure (mmHg)_T1	0.16
45	SvcO2 (%)_T1	0.16
46	Platelets (10^3/mm^3)_T1	0.15
47	Lympho abs count_T2	0.15
48	aPTT_T1	0.14
49	Na (mmol/L)_T2	0.14
50	Pulmonary artery systolic pressure (TR jet by CW + CVP) (mmHg)_T1	0.13

51	Inferior vena cava distensibility index (%)_T2	0.13
52	Diastolic Blood Pressure (mmHg)_T1	0.13
53	A wave (cm/s)_T1	0.13
54	Glycemia (mg/dL)_T2	0.13
55	Value (%)_T2	0.13
56	SOFA_T1	0.11
57	Heart rate (bpm)_T1	0.1
58	Creat Ur_T2	0.1
59	PaO2/FiO2_T2	0.09
60	Respiratory rate (rpm)_T2	0.09
61	Platelet count_T2	0.09
62	BMI	0.08
63	Chloride_T1	0.08
64	Diastolic Blood Pressure (mmHg)_T2	0.08
65	PaCO2 (mmHg)_T2	0.08
66	Heart rate (bpm)_T2.1	0.08
67	E wave (cm/s)_T2	0.08
68	Sat O2/FiO2_T2	0.07
69	SvcO2 (%)_T2	0.07
70	APACHE II_T2	0.07
71	Tricuspid annular plane systolic excursion (TAPSE) (mm)_T1	0.06
72	SOFA_T2	0.06
73	Height (cm)	0.06
74	WBC abs count_T1	0.05
75	Tricuspid annular plane systolic excursion (TAPSE) (mm)_T2	0.05
76	Pplat_T1	0.05
77	Base Excess (mmol/L)_T2	0.04
78	Neutro abs count_T1	0.04
79	Heart rate (bpm)_T2	0.04
80	Chloride_T2	0.04

Table 7: **T1+T2** dataset selected features and their stability scores for the *RFE* feature selection method.

2.3 UFS + RFE

Rank	Feature name	Score
1	Glasgow Coma Scale_T1	0.62
2	HCO3 (mmol/L)_T1	0.53
3	Glasgow Coma Scale_T2	0.45
4	K Ur_T1	0.44
5	Neutro abs count_T2	0.41
6	APACHE II_T1	0.41
7	Tricuspid regurgitation maximal velocity (by CW) (cm/s)_T1	0.37
8	Respiratory rate_T1	0.37
9	Respiratory rate_T2	0.31
10	SOFA_T1	0.28
11	Respiratory rate (rpm)_T1	0.27
12	Platelet count_T1	0.27
13	K Ur_T2	0.23
14	SOFA_T2	0.21
15	Inferior vena cava distensibility index (%)_T1	0.2
16	PaO2 (mmHg)_T2	0.2
17	Sedation Scale (SAS)_T2	0.19
18	Tidal volume (VT)_T1	0.19
19	SvcO2 (%)_T2	0.19
20	Na Ur_T2	0.19
21	Fibrinogen_T1 (g/L)	0.18
22	PaCO2 (mmHg)_T1	0.18
23	Tricuspid annular tissular doppler S wave (DTI) (cm/s)_T1	0.17
24	PT_T2	0.17
25	A wave (cm/s)_T2	0.17
26	Sat O2 (%)_T2	0.16
27	Lactate levels (mmol/L)_T1	0.15
28	PCT_Value (mg/mL)_T1	0.15
29	E wave (cm/s)_T1	0.15
30	Base Excess (mmol/L)_T1	0.14
31	Bilirubin (mg/dL)_T2	0.14
32	APACHE II_T2	0.14
33	PT_T1	0.13
34	Pplat_T1	0.13
35	Neutro abs count_T1	0.12
36	Base Excess (mmol/L)_T2	0.12
37	Value (%)_T1	0.12
38	LVOT average diameter (mm)_T1	0.12
39	HCO3 (mmol/L)_T2	0.11
40	Platelet count_T2	0.11
41	Creat Ur_T2	0.11
42	E wave deceleration time (ms)_T2	0.11
43	* Dobutamine (μg/kg/min)_T1	0.11
44	BMI	0.1
45	LA dilatation by eyeballing_T1	0.1
46	Diastolic Blood Pressure (mmHg)_T1	0.1
47	Na (mmol/L)_T2	0.1
48	PaCO2 (mmHg)_T2	0.1
49	Pulmonary artery systolic pressure (TR jet by CW + CVP) (mmHg)_T1	0.09
50	Lactate levels (mmol/L)_T2	0.09

51	SvcO2 (%)_T1	0.09
52	Weight (kg)	0.09
53	Platelets ($10^3/mm^3$)_T2	0.08
54	Pplat_T2	0.08
55	aPTT_T2	0.07
56	Lympho abs count_T1	0.07
57	Mean arterial pressure (mmHg)_T1	0.07
58	Aortic Valve Regurgitation_T1=No valvular dysfunction	0.07
59	Value (%)_T2	0.07
60	Systolic blood pressure (mmHg)_T1	0.06
61	Heart rate (bpm)_T1	0.06
62	Temperature (°C)_T1	0.06
63	Na Ur_T1	0.06
64	Sedation Scale (SAS)_T1	0.05
65	Sat O2/FiO2_T2	0.05
66	Platelets ($10^3/mm^3$)_T1	0.05
67	Tricuspid annular plane systolic excursion (TAPSE) (mm)_T1	0.05
68	Lympho abs count_T2	0.05
69	Inferior vena cava distensibility index (%)_T2	0.05
70	Mean arterial pressure (mmHg)_T2	0.05
71	Diastolic Blood Pressure (mmHg)_T2	0.05
72	Systolic blood pressure (mmHg)_T2	0.05
73	Hematocrit (%)_T2	0.05
74	A wave (cm/s)_T1	0.05
75	PEEP_T1	0.04
76	Heart rate (bpm)_T2	0.04
77	Bilirubin (mg/dL)_T1	0.04
78	Tidal volume (VT)_T2	0.04
79	PaO2/FiO2_T2	0.04
80	E/e'_T1	0.04

Table 8: **T1+T2** dataset selected features and their stability scores for the *UFS+RFE* feature selection method (the UFS preselected 120 features and the RFE chose the rest).

2.4 Random Forest

Rank	Feature name	Score
1	Lactate levels (mmol/L)_T2	0.54
2	SOFA_T2	0.5
3	Tidal volume (VT)_T2	0.46
4	APACHE II_T1	0.45
5	Urine Output (mL/day)_T1	0.43
6	Urine Output (mL/day)_T2	0.36
7	APACHE II_T2	0.36
8	Respiratory rate_T1	0.33
9	K Ur_T2	0.32
10	SOFA_T1	0.32
11	* Norepinephrine (μg/kg/min)_T2	0.31
12	Neutro abs count_T2	0.29
13	E wave (cm/s)_T1	0.29
14	Na Ur_T2	0.29
15	pH_T2	0.26
16	K Ur_T1	0.26
17	Glasgow Coma Scale_T2	0.25
18	Tricuspid regurgitation maximal velocity (by CW) (cm/s)_T1	0.23
19	PT_T2	0.23
20	Pulmonary artery systolic pressure (TR jet by CW + CVP) (mmHg)_T1	0.22
21	Lactate levels (mmol/L)_T1	0.22
22	PCT_Value (mg/mL)_T1	0.22
23	Respiratory rate (rpm)_T1	0.21
24	Fluid Balance (ml)_T2	0.21
25	Sat O2/FiO2_T2	0.2
26	Temperature (°C)_T2	0.2
27	Platelet count_T1	0.2
28	Creatinine (mg/dL)_T2	0.2
29	Platelet count_T2	0.19
30	E/e'_T1	0.19
31	Glycemia (mg/dL)_T1	0.19
32	Pplat_T2	0.18
33	Neutro abs count_T1	0.18
34	SvcO2 (%)_T2	0.18
35	HCO3 (mmol/L)_T2	0.17
36	PEEP_T1	0.17
37	Tidal volume (VT)_T1	0.17
38	Lympho abs count_T2	0.17
39	LA dilatation by eyeballing_T1	0.17
40	* Dobutamine (μg/kg/min)_T1	0.17
41	Sedation Scale (SAS)_T2	0.16
42	Pplat_T1	0.16
43	Fluid Balance (ml)_T1	0.16
44	E wave deceleration time (ms)_T2	0.16
45	FiO2_T2	0.15
46	Temperature (°C)_T1	0.15
47	pH_T1	0.14
48	* Norepinephrine (μg/kg/min)_T1	0.14
49	Heart rate (bpm)_T1	0.14
50	Na Ur_T1	0.14

51	Prothrombin time (INR)_T2	0.13
52	aPTT_T1	0.13
53	Sat O2 (%)_T2	0.13
54	RBC count_T1	0.13
55	Fibrinogen_T1 (g/L)	0.13
56	Creat Ur_T2	0.13
57	A wave (cm/s)_T1	0.13
58	Glasgow Coma Scale_T1	0.13
59	Troponine_T2	0.13
60	WBC abs count_T1	0.12
61	PaCO2 (mmHg)_T1	0.12
62	PaCO2 (mmHg)_T2	0.12
63	Midazolam (μg/kg/min)_T1	0.12
64	PEEP_T2	0.11
65	PaO2/FiO2_T1	0.11
66	RBC count_T2	0.11
67	Respiratory rate_T2	0.11
68	SvcO2 (%)_T1	0.11
69	Base Excess (mmol/L)_T2	0.11
70	Lateral e' (cm/s)_T1	0.11
71	PaO2/FiO2_T2	0.1
72	BMI	0.1
73	Lympho abs count_T1	0.1
74	Inferior vena cava distensibility index (%)_T1	0.1
75	Chloride_T1	0.1
76	K (mmol/L)_T2	0.1
77	Na (mmol/L)_T2	0.1
78	Base Excess (mmol/L)_T1	0.1
79	Creat Ur_T1	0.1
80	aPTT_T2	0.08

Table 9: **T1+T2** dataset selected features and their stability scores for the *Random Forest* feature selection method.

2.5 Aggregated features

Rank	Feature name	Score
1	APACHE II_T1	0.5875
2	SOFA_T2	0.4425
3	Tricuspid regurgitation maximal velocity (by CW) (cm/s)_T1	0.3425
4	Glasgow Coma Scale_T1	0.33
5	Neutro abs count_T2	0.32
6	Glasgow Coma Scale_T2	0.32
7	Platelet count_T1	0.2975
8	K Ur_T1	0.2975
9	APACHE II_T2	0.2975
10	SOFA_T1	0.2775
11	Respiratory rate_T1	0.27
12	HCO3 (mmol/L)_T1	0.2575
13	Lactate levels (mmol/L)_T2	0.2425
14	E wave (cm/s)_T1	0.23
15	Tidal volume (VT)_T1	0.23
16	Respiratory rate (rpm)_T1	0.225
17	K Ur_T2	0.205
18	Na Ur_T2	0.1875
19	PT_T2	0.185
20	LA dilatation by eyeballing_T1	0.18
21	Respiratory rate_T2	0.18
22	Tidal volume (VT)_T2	0.1725
23	Pulmonary artery systolic pressure (TR jet by CW + CVP) (mmHg)_T1	0.16
24	PT_T1	0.1575
25	PCT_Value (mg/mL)_T1	0.155
26	* Norepinephrine (μg/kg/min)_T2	0.1525
27	Leukocytes (total*1000/mm^3)_T2	0.1475
28	PaO2 (mmHg)_T2	0.145
29	Heart rate (bpm)_T1.1	0.145
30	Base Excess (mmol/L)_T1	0.1425
31	Sedation Scale (SAS)_T2	0.14
32	Inferior vena cava distensibility index (%)_T1	0.14
33	A wave (cm/s)_T2	0.1375
34	Platelet count_T2	0.135
35	pH_T2	0.1325
36	Urine Output (mL/day)_T1	0.1325
37	Na Ur_T1	0.125
38	PaCO2 (mmHg)_T1	0.125
39	SvcO2 (%)_T2	0.1225
40	Hematocrit (%)_T1	0.1175
41	Value (%)_T1	0.1175
42	Sat O2/FiO2_T2	0.115
43	Urine Output (mL/day)_T2	0.1125
44	E wave deceleration time (ms)_T2	0.1125
45	Tricuspid annular tissular doppler S wave (DTI) (cm/s)_T1	0.11
46	Na (mmol/L)_T1	0.105
47	Weight (kg)	0.105
48	Lactate levels (mmol/L)_T1	0.1025
49	PaO2 (mmHg)_T1	0.1025
50	Platelets (10^3/mm^3)_T2	0.1

51	FiO2_T2	0.1
52	SvcO2 (%)_T1	0.1
53	* Norepinephrine (μg/kg/min)_T1	0.1
54	E wave deceleration time (ms)_T1	0.1
55	Lympho abs count_T2	0.095
56	Pplat_T1	0.095
57	Sat O2/FiO2_T1	0.0925
58	Hematocrit (%)_T2	0.0925
59	Creat Ur_T2	0.0925
60	aPTT_T2	0.09
61	Creatinine (mg/dL)_T2	0.09
62	PaO2/FiO2_T1	0.0875
63	Neutro abs count_T1	0.0875
64	HCO3 (mmol/L)_T2	0.085
65	Na (mmol/L)_T2	0.085
66	LVOT average diameter (mm)_T1	0.085
67	Creat Ur_T1	0.0825
68	* Dobutamine (μg/kg/min)_T1	0.0825
69	Fibrinogen_T1 (g/L)	0.08
70	A wave (cm/s)_T1	0.08
71	WBC abs count_T2	0.0775
72	aPTT_T1	0.075
73	Heart rate (bpm)_T1	0.075
74	CRP_Value (mg/L)_T2	0.075
75	PaCO2 (mmHg)_T2	0.075
76	Base Excess (mmol/L)_T2	0.0725
77	Systolic blood pressure (mmHg)_T1	0.0725
78	Sat O2 (%)_T2	0.0725
79	Diastolic Blood Pressure (mmHg)_T1	0.0725
80	CRP_Value (mg/L)_T1	0.065

Table 10: **T1+T2** dataset aggregated selected features and their stability scores for all feature selection methods.

3 The Full dataset

3.1 Univariate feature selection

Rank	Feature name	Score
1	Is the patient under other drugs_T2	0.12
2	Was shock diagnosed within 48 from hospital admission	0.12
3	Fibrinogen_T2 (g/L)	0.11
4	Lactate levels (mmol/L)_T3	0.1
5	Lympho abs count_T2	0.1
6	Fibrinogen_T3 (g/L)	0.1
7	K (mmol/L)_T1	0.1
8	pH_T2	0.09
9	PT_T2	0.09
10	Is the patient affected by Prolonged Arrhythmias (PA)_T2	0.09
11	FiO2_T1	0.09
12	PaO2 (mmHg)_T2	0.09
13	Tricuspid Valve Regurgitation_T3=No valvular dysfunction	0.09
14	Tricuspid Valve Regurgitation_T1=No valvular dysfunction	0.09
15	Tricuspid annular plane systolic excursion (TAPSE) (mm)_T2	0.09
16	Fluid Balance (ml)_T1	0.09
17	Na (mmol/L)_T3	0.09
18	Respiratory rate_T1	0.09
19	Site of sampling=Abdominal drain	0.08
20	Sedation Scale (SAS)_T2	0.08
21	Bilirubin (mg/dL)_T3	0.08
22	Temperature (°C)_T3	0.08
23	Pulmonary artery systolic pressure (TR jet by CW + CVP) (mmHg)_T1	0.08
24	Is the patient affected by Acute Myocardial Infarction (AMI)_T1	0.08
25	Hypotension (SBP $<$ 90 mmHg or MAP $<$ 70 mmHg or SBP decrease $>$ 40 mmHg)_T1	0.08
26	WBC abs count_T1	0.08
27	PaO2 (mmHg)_T3	0.08
28	E wave deceleration time (ms)_T3	0.08
29	Cardiac output (ml/min)_T3	0.08
30	SOFA_T2	0.08
31	Site of sampling=Respiratory tract	0.08
32	Chloride_T1	0.08
33	Na (mmol/L)_T1	0.08
34	A wave (cm/s)_T1	0.08
35	Leukocytes (total*1000/mm^3)_T2	0.08
36	Is the patient under other drugs_T1	0.08
37	Sat O2/FiO2_T3	0.08
38	Systolic blood pressure (mmHg)_T2	0.08
39	Sat O2 (%)_T1	0.08
40	Tricuspid Valve Regurgitation_T2=Mild	0.08
41	pH_T3	0.07
42	pH_T1	0.07
43	* Norepinephrine (μg/kg/min)_T1	0.07
44	Mean arterial pressure (mmHg)_T2	0.07
45	Was the patient transfused_T3	0.07
46	Mode_T2=Volume controlled	0.07
47	LV Hypertrophy (LV mass)_T3=Moderate	0.07
48	Base Excess (mmol/L)_T3	0.07
49	Glycemia (mg/dL)_T1	0.07
50	Tricuspid Valve Regurgitation_T3=Mild	0.07

51	Mitral Valve Regurgitation_T2=No valvular dysfunction	0.07
52	Heart rate (bpm)_T3	0.07
53	Tidal volume (VT)_T2	0.07
54	Sample 1=Positive	0.07
55	LV Dilatation (LVEDV)_T3=Mild	0.07
56	Fibrinogen_T1 (g/L)	0.07
57	Creat Ur_T2	0.07
58	Tricuspid Valve Regurgitation_T2=No valvular dysfunction	0.07
59	LVOT average velocity-time integral (cm)_T1	0.07
60	Tracheal Intubation_T1	0.07
61	Site of sampling.1=Urine	0.07
62	Respiratory rate (rpm)_T1	0.07
63	Respiratory rate (rpm)_T2	0.07
64	Sample 1=Negative	0.07
65	Fluid Balance (ml)_T2	0.07
66	Acute Kidney Injury_T1=AKIN II	0.07
67	K Ur_T1	0.07
68	Hematocrit (%)_T2	0.07
69	Mitral Valve Regurgitation_T2=Mild	0.07
70	A wave (cm/s)_T2	0.07
71	Leukocytes (total*1000/mm^3)_T3	0.07
72	Leukocytes (total*1000/mm^3)_T1	0.07
73	Heart rate (bpm)_T1.1	0.07
74	Is the patient under other drugs_T3	0.07
75	HCO3 (mmol/L)_T1	0.07
76	Sat O2/FiO2_T2	0.07
77	Sat O2/FiO2_T1	0.07
78	Tricuspid Valve Regurgitation_T1=Moderate	0.07
79	Site of sampling=CSF	0.07
80	Site of sampling.1=Respiratory tract	0.07

Table 11: **Full** dataset selected features and their stability scores for the *UFS* feature selection.

3.2 Recursive feature elimination

Rank	Feature name	Score
1	Heart rate (bpm)_T1	0.32
2	Platelet count_T3	0.32
3	Systolic blood pressure (mmHg)_T2	0.3
4	A wave (cm/s)_T2	0.3
5	aPTT_T1	0.29
6	Weight (kg)	0.29
7	PT_T1	0.28
8	Creat Ur_T1	0.27
9	E wave deceleration time (ms)_T1	0.27
10	Systolic blood pressure (mmHg)_T1	0.26
11	A wave (cm/s)_T1	0.26
12	Tricuspid regurgitation maximal velocity (by CW) (cm/s)_T1	0.25
13	PaO2 (mmHg)_T1	0.25
14	Height (cm)	0.25
15	CRP_Value (mg/L)_T3	0.24
16	Na Ur_T1	0.24
17	Glycemia (mg/dL)_T3	0.24
18	E wave deceleration time (ms)_T3	0.24
19	PaO2/FiO2_T3	0.23
20	Heart rate (bpm)_T2	0.22
21	Platelet count_T1	0.22
22	Platelet count_T2	0.22
23	PaO2 (mmHg)_T2	0.22
24	PaCO2 (mmHg)_T1	0.22
25	E wave (cm/s)_T2	0.22
26	E wave (cm/s)_T1	0.22
27	PCT_Value (mg/mL)_T1	0.21
28	Glycemia (mg/dL)_T2	0.21
29	Diastolic Blood Pressure (mmHg)_T1	0.2
30	E wave deceleration time (ms)_T2	0.2
31	Na Ur_T2	0.2
32	Heart rate (bpm)_T1.1	0.2
33	Base Excess (mmol/L)_T1	0.19
34	Systolic blood pressure (mmHg)_T3	0.19
35	WBC abs count_T2	0.19
36	Respiratory rate (rpm)_T1	0.19
37	Respiratory rate (rpm)_T2	0.19
38	PT_T2	0.19
39	K Ur_T1	0.19
40	Sat O2/FiO2_T3	0.18
41	Mean arterial pressure (mmHg)_T1	0.18
42	Hematocrit (%)_T2	0.18
43	Glycemia (mg/dL)_T1	0.18
44	Glasgow Coma Scale_T1	0.18
45	HCO3 (mmol/L)_T1	0.17
46	Platelets ($10^3/mm^3$)_T3	0.17
47	Heart rate (bpm)_T3	0.17
48	aPTT_T2	0.17
49	Tidal volume (VT)_T1	0.17
50	Platelets ($10^3/mm^3$)_T1	0.17

51	Leukocytes (total*1000/mm^3)_T2	0.17
52	Leukocytes (total*1000/mm^3)_T1	0.17
53	Heart rate (bpm)_T2.1	0.17
54	Value (%)_T2	0.17
55	Sat O2/FiO2_T1	0.16
56	PaO2/FiO2_T1	0.16
57	Chloride_T1	0.16
58	A wave (cm/s)_T3	0.16
59	E wave (cm/s)_T3	0.16
60	BMI	0.15
61	Platelets (10^3/mm^3)_T2	0.15
62	PT_T3	0.15
63	PaO2 (mmHg)_T3	0.15
64	Diastolic Blood Pressure (mmHg)_T2	0.15
65	Heart rate (bpm)_T3.1	0.15
66	Na (mmol/L)_T2	0.15
67	Creat Ur_T2	0.15
68	Tidal volume (VT)_T2	0.14
69	CRP_Value (mg/L)_T2	0.14
70	Mean arterial pressure (mmHg)_T2	0.14
71	K Ur_T2	0.14
72	PaCO2 (mmHg)_T2	0.14
73	PaO2/FiO2_T2	0.13
74	Neutro abs count_T2	0.13
75	Value (%)_T3	0.13
76	Sat O2/FiO2_T2	0.12
77	WBC abs count_T1	0.12
78	CRP_Value (mg/L)_T1	0.12
79	Respiratory rate_T1	0.12
80	E/A ratio_T2	0.12

Table 12: **Full** dataset selected features and their stability scores for the *RFE* feature selection method.

3.3 UFS + RFE

Rank	Feature name	Score
1	Height (cm)	0.21
2	Respiratory rate (rpm)_T1	0.19
3	K Ur_T1	0.17
4	Hematocrit (%)_T2	0.17
5	Na (mmol/L)_T1	0.17
6	PaCO2 (mmHg)_T1	0.17
7	WBC abs count_T1	0.16
8	PaCO2 (mmHg)_T2	0.16
9	HCO3 (mmol/L)_T1	0.15
10	Tricuspid annular plane systolic excursion (TAPSE) (mm)_T2	0.15
11	Inferior vena cava distensibility index (%)_T1	0.15
12	SvcO2 (%)_T1	0.15
13	APACHE II_T2	0.15
14	LVOT average velocity-time integral (cm)_T1	0.15
15	E wave (cm/s)_T2	0.15
16	E wave (cm/s)_T3	0.15
17	Glasgow Coma Scale_T2	0.15
18	Systolic blood pressure (mmHg)_T2	0.14
19	Respiratory rate (rpm)_T3	0.14
20	PaO2 (mmHg)_T3	0.14
21	Respiratory rate_T2	0.14
22	E/e'_T1	0.14
23	E/e'_T2	0.14
24	Weight (kg)	0.14
25	HCO3 (mmol/L)_T3	0.13
26	aPTT_T3	0.13
27	Respiratory rate_T1	0.13
28	A wave (cm/s)_T1	0.13
29	BMI	0.12
30	Base Excess (mmol/L)_T1	0.12
31	PT_T1	0.12
32	Systolic blood pressure (mmHg)_T1	0.12
33	Sat O2 (%)_T1	0.12
34	PT_T2	0.12
35	Mean arterial pressure (mmHg)_T1	0.12
36	Heart rate (bpm)_T1	0.12
37	Heart rate (bpm)_T3.1	0.12
38	Value (%)_T1	0.12
39	Value (%)_T3	0.12
40	A wave (cm/s)_T2	0.12
41	Pplat_T1	0.12
42	E wave deceleration time (ms)_T3	0.12
43	Glasgow Coma Scale_T1	0.12
44	Midazolam (μg/kg/min)_T1	0.12
45	Systolic blood pressure (mmHg)_T3	0.11
46	Sat O2 (%)_T3	0.11
47	Sat O2 (%)_T2	0.11
48	Neutro abs count_T1	0.11
49	Bilirubin (mg/dL)_T1	0.11
50	Tricuspid annular plane systolic excursion (TAPSE) (mm)_T3	0.11

51	PT_T3	0.11
52	PaO2 (mmHg)_T2	0.11
53	Base Excess (mmol/L)_T3	0.11
54	Diastolic Blood Pressure (mmHg)_T2	0.11
55	Diastolic Blood Pressure (mmHg)_T3	0.11
56	Na (mmol/L)_T2	0.11
57	Leukocytes (total*1000/mm^3)_T3	0.11
58	Leukocytes (total*1000/mm^3)_T1	0.11
59	Glasgow Coma Scale_T3	0.11
60	* Dobutamine (μg/kg/min)_T1	0.11
61	PCT_Value (mg/mL)_T1	0.1
62	Chloride_T1	0.1
63	Inferior vena cava distensibility index (%)_T2	0.1
64	Diastolic Blood Pressure (mmHg)_T1	0.1
65	SvcO2 (%)_T2	0.1
66	Hematocrit (%)_T1	0.1
67	Fibrinogen_T1 (g/L)	0.1
68	SOFA_T3	0.1
69	APACHE II_T3	0.1
70	Creat Ur_T2	0.1
71	Base Excess (mmol/L)_T2	0.1
72	LVOT average velocity-time integral (cm)_T2	0.1
73	Glycemia (mg/dL)_T3	0.1
74	Leukocytes (total*1000/mm^3)_T2	0.1
75	Lateral e' (cm/s)_T2	0.1
76	PaCO2 (mmHg)_T3	0.1
77	Pplat_T2	0.1
78	HCO3 (mmol/L)_T2	0.09
79	Pulmonary artery systolic pressure (TR jet by CW + CVP) (mmHg)_T1	0.09
80	PEEP_T2	0.09

Table 13: **Full** dataset selected features and their stability scores for the *UFS+RFE* feature selection method (the UFS preselected 120 features and the RFE chose the rest).

3.4 Random Forest

Rank	Feature name	Score
1	Neutro abs count_T1	0.2
2	Platelets ($10^3/mm^3$)_T3	0.19
3	E/e'_T2	0.18
4	Lateral e' (cm/s)_T2	0.18
5	Creat Ur_T2	0.17
6	Urine Output (mL/day)_T2	0.17
7	Lateral e' (cm/s)_T1	0.17
8	Respiratory rate (rpm)_T1	0.16
9	Lympho abs count_T1	0.16
10	Mean arterial pressure (mmHg)_T1	0.16
11	Diastolic Blood Pressure (mmHg)_T2	0.16
12	E wave deceleration time (ms)_T2	0.16
13	Tricuspid annular tissular doppler S wave (DTI) (cm/s)_T1	0.15
14	Sat O2/FiO2_T2	0.15
15	Platelets ($10^3/mm^3$)_T1	0.15
16	FiO2_T3	0.15
17	Tidal volume (VT)_T1	0.15
18	RBC count_T3	0.15
19	Fluid Balance (ml)_T2	0.15
20	Platelet count_T2	0.15
21	Pulmonary artery systolic pressure (TR jet by CW + CVP) (mmHg)_T1	0.15
22	Hematocrit (%)_T1	0.15
23	Na Ur_T1	0.15
24	Base Excess (mmol/L)_T1	0.15
25	aPTT_T3	0.15
26	Troponine_T1	0.15
27	pH_T2	0.14
28	Base Excess (mmol/L)_T2	0.14
29	Heart rate (bpm)_T2.1	0.14
30	Fluid Balance (ml)_T1	0.14
31	Lympho abs count_T2	0.14
32	CRP_Value (mg/L)_T1	0.14
33	Diastolic Blood Pressure (mmHg)_T1	0.14
34	K Ur_T2	0.14
35	Creat Ur_T1	0.14
36	Fibrinogen_T1 (g/L)	0.14
37	Urine Output (mL/day)_T1	0.14
38	A wave (cm/s)_T2	0.14
39	E/e'_T1	0.14
40	Glycemia (mg/dL)_T1	0.14
41	Leukocytes (total*1000/mm^3)_T1	0.14
42	Sat O2/FiO2_T3	0.13
43	* Norepinephrine (μg/kg/min)_T1	0.13
44	Fibrinogen_T2 (g/L)	0.13
45	Lympho abs count_T3	0.13
46	PaO2 (mmHg)_T1	0.13
47	Mean arterial pressure (mmHg)_T3	0.13
48	Diastolic Blood Pressure (mmHg)_T3	0.13
49	Hematocrit (%)_T3	0.13
50	Na Ur_T2	0.13

51	SOFA_T3	0.13
52	LVOT average velocity-time integral (cm)_T1	0.13
53	Glycemia (mg/dL)_T2	0.13
54	Leukocytes (total*1000/mm^3)_T3	0.13
55	E wave (cm/s)_T3	0.13
56	Midazolam (μg/kg/min)_T1	0.13
57	pH_T3	0.12
58	Temperature (°C)_T3	0.12
59	aPTT_T1	0.12
60	Heart rate (bpm)_T3	0.12
61	Heart rate (bpm)_T1	0.12
62	WBC abs count_T2	0.12
63	RBC count_T1	0.12
64	Respiratory rate (rpm)_T3	0.12
65	Inferior vena cava distensibility index (%)_T2	0.12
66	E/A ratio_T2	0.12
67	Hematocrit (%)_T2	0.12
68	K (mmol/L)_T3	0.12
69	A wave (cm/s)_T3	0.12
70	APACHE II_T2	0.12
71	A wave (cm/s)_T1	0.12
72	Platelets (10^3/mm^3)_T2	0.12
73	Lateral e' (cm/s)_T3	0.12
74	E wave deceleration time (ms)_T1	0.12
75	Weight (kg)	0.12
76	HCO3 (mmol/L)_T2	0.11
77	Cardiac output (ml/min)_T1	0.11
78	Prothrombin time (INR)_T3	0.11
79	Lactate levels (mmol/L)_T2	0.11
80	Systolic blood pressure (mmHg)_T1	0.11

Table 14: **Full** dataset selected features and their stability scores for the *Random Forest* feature selection method.

3.5 Aggregated features

Rank	Feature name	Score
1	A wave (cm/s)_T2	0.1575
2	Systolic blood pressure (mmHg)_T2	0.1575
3	Heart rate (bpm)_T1	0.1525
4	Respiratory rate (rpm)_T1	0.1525
5	Weight (kg)	0.15
6	Height (cm)	0.1475
7	A wave (cm/s)_T1	0.1475
8	PT_T1	0.1375
9	E wave deceleration time (ms)_T3	0.135
10	Hematocrit (%)_T2	0.135
11	Platelet count_T3	0.135
12	Creat Ur_T1	0.1325
13	E wave deceleration time (ms)_T1	0.1325
14	PaCO2 (mmHg)_T1	0.13
15	K Ur_T1	0.13
16	E wave (cm/s)_T2	0.13
17	aPTT_T1	0.1275
18	Mean arterial pressure (mmHg)_T1	0.125
19	Base Excess (mmol/L)_T1	0.125
20	PaO2 (mmHg)_T2	0.125
21	Systolic blood pressure (mmHg)_T1	0.125
22	PT_T2	0.1225
23	Glycemia (mg/dL)_T3	0.1225
24	Creat Ur_T2	0.1225
25	Platelet count_T2	0.1225
26	Leukocytes (total*1000/mm^3)_T1	0.1225
27	HCO3 (mmol/L)_T1	0.1225
28	Glycemia (mg/dL)_T2	0.12
29	PaO2 (mmHg)_T1	0.12
30	Diastolic Blood Pressure (mmHg)_T1	0.12
31	E wave (cm/s)_T3	0.12
32	WBC abs count_T1	0.1175
33	Tricuspid regurgitation maximal velocity (by CW) (cm/s)_T1	0.1175
34	Diastolic Blood Pressure (mmHg)_T2	0.1175
35	PCT_Value (mg/mL)_T1	0.115
36	Na Ur_T1	0.115
37	Leukocytes (total*1000/mm^3)_T2	0.115
38	Platelets (10^3/mm^3)_T1	0.1125
39	Platelets (10^3/mm^3)_T3	0.1125
40	PaO2 (mmHg)_T3	0.1125
41	LVOT average velocity-time integral (cm)_T1	0.1125
42	Respiratory rate (rpm)_T2	0.1125
43	Platelet count_T1	0.1125
44	Sat O2/FiO2_T3	0.1125
45	Glycemia (mg/dL)_T1	0.11
46	Heart rate (bpm)_T3	0.11
47	Systolic blood pressure (mmHg)_T3	0.11
48	Na (mmol/L)_T1	0.11
49	E wave deceleration time (ms)_T2	0.11
50	Heart rate (bpm)_T1.1	0.11

51	Heart rate (bpm)_T2	0.11
52	WBC abs count_T2	0.1075
53	Neutro abs count_T1	0.1075
54	Chloride_T1	0.1075
55	K Ur_T2	0.1075
56	Na Ur_T2	0.105
57	PaCO2 (mmHg)_T2	0.105
58	Tidal volume (VT)_T1	0.105
59	APACHE II_T2	0.105
60	A wave (cm/s)_T3	0.105
61	Heart rate (bpm)_T3.1	0.1025
62	E wave (cm/s)_T1	0.1025
63	PT_T3	0.1
64	Pulmonary artery systolic pressure (TR jet by CW + CVP) (mmHg)_T1	0.1
65	PaO2/FiO2_T3	0.1
66	Hematocrit (%)_T1	0.1
67	E/e'_T2	0.1
68	SvcO2 (%)_T1	0.0975
69	Heart rate (bpm)_T2.1	0.0975
70	BMI	0.0975
71	Lympho abs count_T2	0.0975
72	Respiratory rate (rpm)_T3	0.0975
73	Leukocytes (total*1000/mm^3)_T3	0.0975
74	aPTT_T3	0.0975
75	Respiratory rate_T1	0.0975
76	Mean arterial pressure (mmHg)_T2	0.095
77	PaCO2 (mmHg)_T3	0.095
78	Platelets (10^3/mm^3)_T2	0.095
79	CRP_Value (mg/L)_T3	0.095
80	CRP_Value (mg/L)_T2	0.095

Table 15: **Full** dataset aggregated selected features and their stability scores for all feature selection methods.

4 Additional causal Bayesian networks

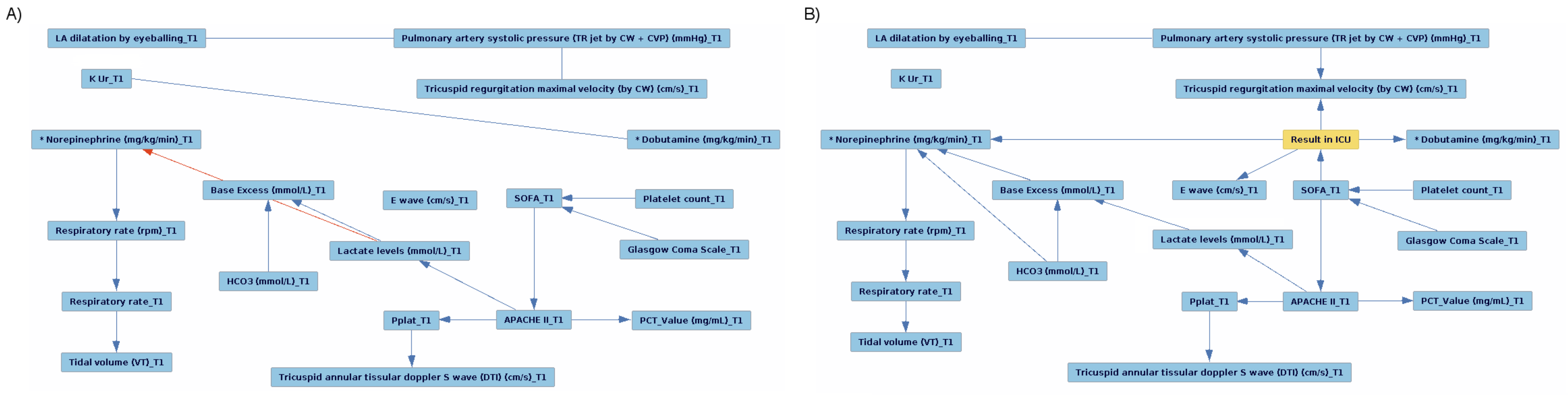

Figure 1: **The *(T1, Aggr.)* *CBN*.** The CBN for the *(T1, Aggr.)* feature set obtained: a) without the target feature; b) with the target feature.

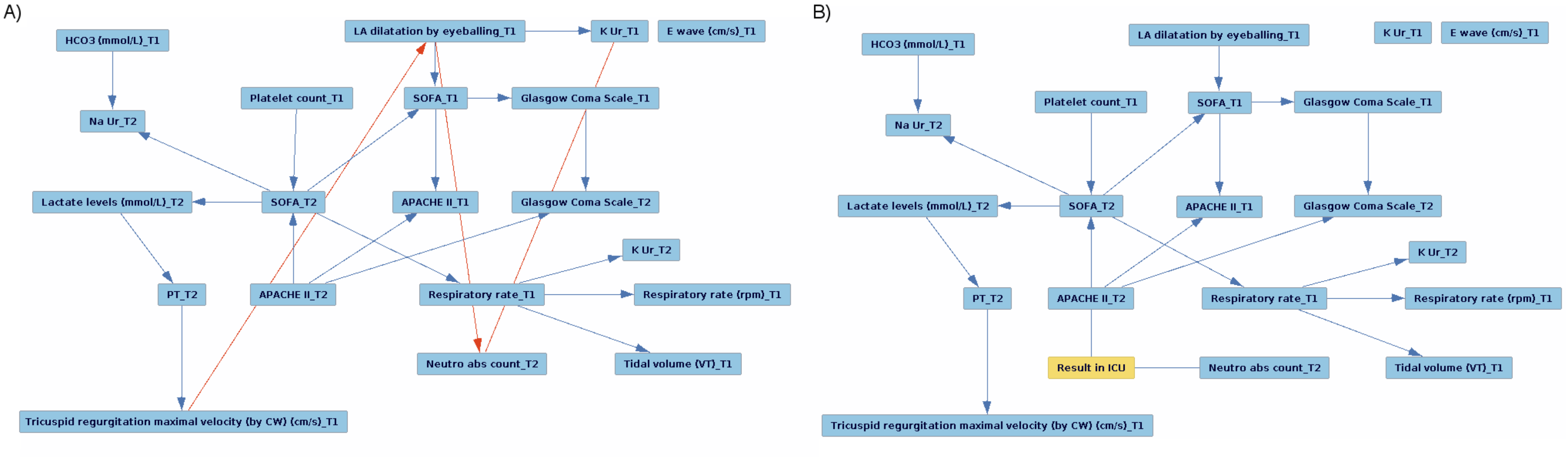

Figure 2: **The *(T1+T2, Aggr.)* CBN.** The CBN for the *(T1+T2, Aggr.)* feature set obtained: a) without the target feature; b) with the target feature.

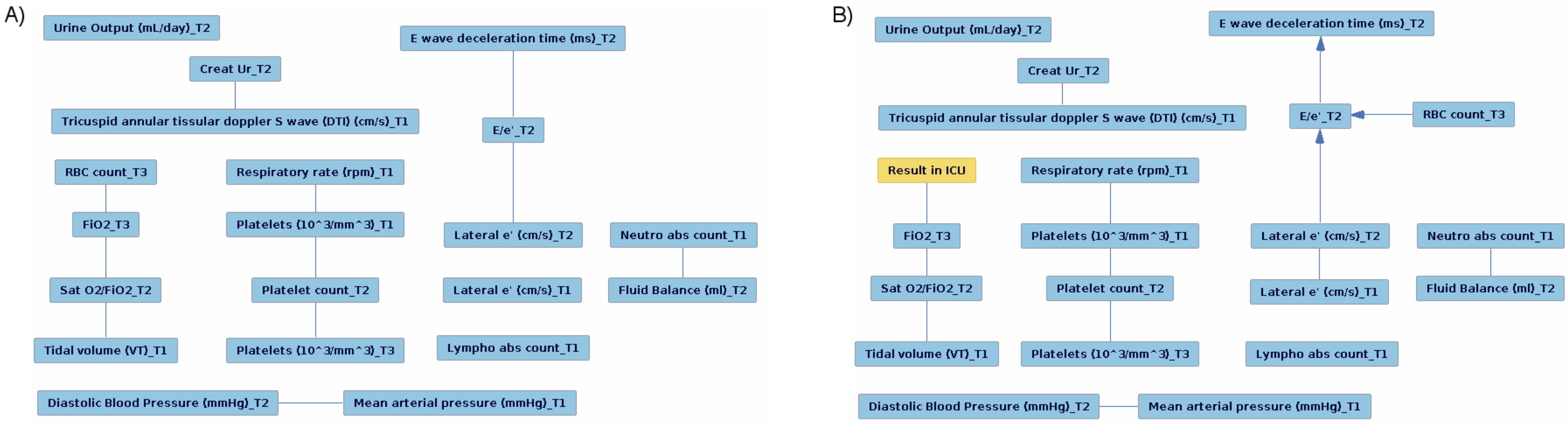

Figure 3: **The *(Full, RF)* CBN.** The CBN for the *(Full, RF)* feature set obtained: a) without the target feature; b) with the target feature.

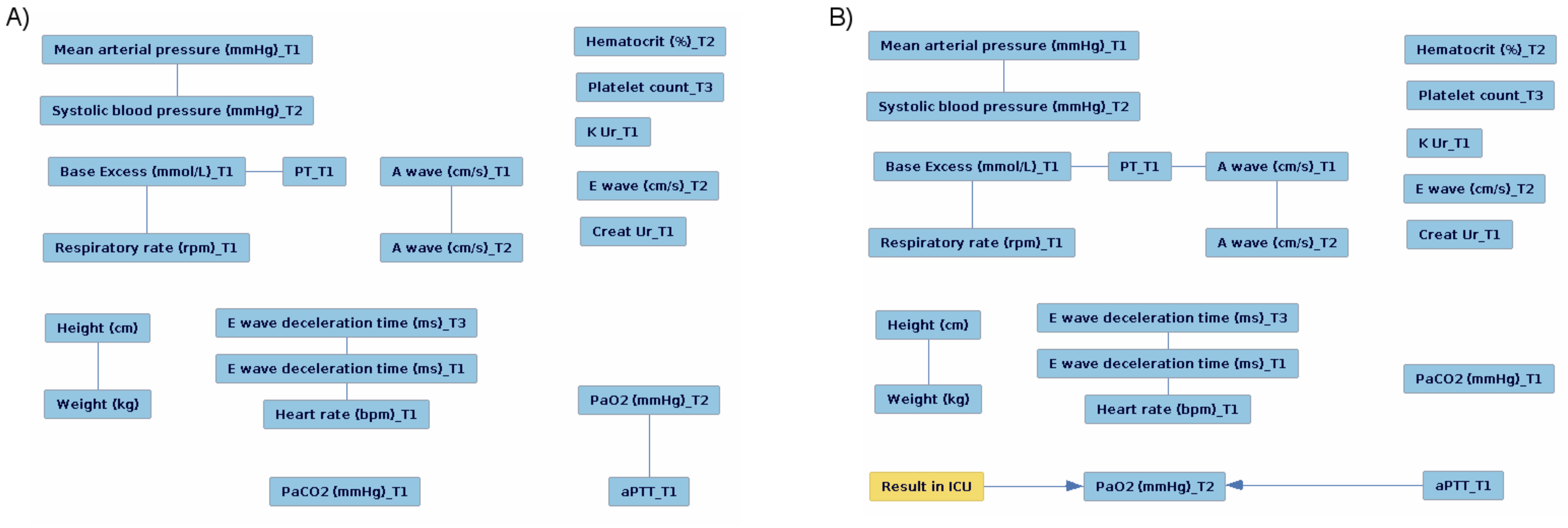

Figure 4: **The *(Full, Aggr.)* CBN.** The CBN for the *(Full, Aggr.)* feature set obtained: a) without the target feature; b) with the target feature.

5 Full list of ShockOmics attributes

Index	Name of the feature
1	ID
2	Gender
3	Weight (kg)
4	Height (cm)
5	BMI
6	Reason for Admission
7	ICU Admission
8	Which type of shock
9	Was shock diagnosed within 48 from hospital admission
10	Number of affected organs
11	Hypotension (SBP $<$ 90 mmHg or MAP $<$ 70 mmHg or SBP decrease $>$ 40 mmHg)_-T1
12	Hypotension (SBP $<$ 90 mmHg or MAP $<$ 70 mmHg or SBP decrease $>$ 40 mmHg)_-T2
13	Hypotension (SBP $<$ 90 mmHg or MAP $<$ 70 mmHg or SBP decrease $>$ 40 mmHg)_-T3
14	Lactate levels (mmol/L)_T1
15	Lactate levels (mmol/L)_T2
16	Lactate levels (mmol/L)_T3
17	SvcO2 (%)_T1
18	SvcO2 (%)_T2
19	SvcO2 (%)_T3
20	SvO2 (%)_T1
21	SvO2 (%)_T2
22	SvO2 (%)_T3
23	Is there an active infection
24	Is the patient affected by Acute Myocardial Infarction (AMI)_T1
25	Is the patient affected by Acute Myocardial Infarction (AMI)_T2
26	Is the patient affected by Acute Myocardial Infarction (AMI)_T3
27	Is the patient affected by Prolonged Arrhythmias (PA)_T1
28	Is the patient affected by Prolonged Arrhythmias (PA)_T2
29	Is the patient affected by Prolonged Arrhythmias (PA)_T3
30	Was the patient transfused_T1
31	Was the patient transfused_T2
32	Was the patient transfused_T3
33	Rhythm_T1
34	Rhythm_T2
35	Rhythm_T3
36	LV Hypertrophy (LV mass)_T1
37	LV Hypertrophy (LV mass)_T2
38	LV Hypertrophy (LV mass)_T3
39	LV Dilatation (LVEDV)_T1
40	LV Dilatation (LVEDV)_T2
41	LV Dilatation (LVEDV)_T3
42	Segmental LV wall kinetics_T1
43	Segmental LV wall kinetics_T2
44	Segmental LV wall kinetics_T3
45	Segments affected by eyeballing_T1
46	Segments affected by eyeballing_T2
47	Segments affected by eyeballing_T3
48	LVOT average diameter (mm)_T1
49	LVOT average diameter (mm)_T2
50	LVOT average diameter (mm)_T3

51	LVOT average velocity-time integral (cm)_T1
52	LVOT average velocity-time integral (cm)_T2
53	LVOT average velocity-time integral (cm)_T3
54	Heart rate (bpm)_T1
55	Heart rate (bpm)_T2
56	Heart rate (bpm)_T3
57	Cardiac output (ml/min)_T1
58	Cardiac output (ml/min)_T2
59	Cardiac output (ml/min)_T3
60	Value (%)_T1
61	Value (%)_T2
62	Value (%)_T3
63	LA dilatation by eyeballing_T1
64	LA dilatation by eyeballing_T2
65	LA dilatation by eyeballing_T3
66	E wave (cm/s)_T1
67	E wave (cm/s)_T2
68	E wave (cm/s)_T3
69	E wave deceleration time (ms)_T1
70	E wave deceleration time (ms)_T2
71	E wave deceleration time (ms)_T3
72	A wave (cm/s)_T1
73	A wave (cm/s)_T2
74	A wave (cm/s)_T3
75	E/A ratio_T1
76	E/A ratio_T2
77	E/A ratio_T3
78	Lateral e' (cm/s)_T1
79	Lateral e' (cm/s)_T2
80	Lateral e' (cm/s)_T3
81	E/e'_T1
82	E/e'_T2
83	E/e'_T3
84	Tricuspid annular plane systolic excursion (TAPSE) (mm)_T1
85	Tricuspid annular plane systolic excursion (TAPSE) (mm)_T2
86	Tricuspid annular plane systolic excursion (TAPSE) (mm)_T3
87	Tricuspid annular tissular doppler S wave (DTI) (cm/s)_T1
88	Tricuspid annular tissular doppler S wave (DTI) (cm/s)_T2
89	Tricuspid annular tissular doppler S wave (DTI) (cm/s)_T3
90	Tricuspid regurgitation maximal velocity (by CW) (cm/s)_T1
91	Tricuspid regurgitation maximal velocity (by CW) (cm/s)_T2
92	Tricuspid regurgitation maximal velocity (by CW) (cm/s)_T3
93	Pulmonary artery systolic pressure (TR jet by CW + CVP) (mmHg)_T1
94	Pulmonary artery systolic pressure (TR jet by CW + CVP) (mmHg)_T2
95	Pulmonary artery systolic pressure (TR jet by CW + CVP) (mmHg)_T3
96	RV area/LV area_T1
97	RV area/LV area_T2
98	RV area/LV area_T3
99	RV cross section D shape_T1
100	RV cross section D shape_T2

101	RV cross section D shape_T3
102	Paradoxical interventricular septum movements_T1
103	Paradoxical interventricular septum movements_T2
104	Paradoxical interventricular septum movements_T3
105	Aortic Valve Regurgitation_T1
106	Aortic Valve Regurgitation_T2
107	Aortic Valve Regurgitation_T3
108	Aortic Valve Stenosis_T1
109	Aortic Valve Stenosis_T2
110	Aortic Valve Stenosis_T3
111	Mitral Valve Regurgitation_T1
112	Mitral Valve Regurgitation_T2
113	Mitral Valve Regurgitation_T3
114	Mitral Valve Stenosis_T1
115	Mitral Valve Stenosis_T2
116	Mitral Valve Stenosis_T3
117	Tricuspid Valve Regurgitation_T1
118	Tricuspid Valve Regurgitation_T2
119	Tricuspid Valve Regurgitation_T3
120	LVOT VTI or SV variation (%)_T1
121	Inferior vena cava distensibility index (%)_T1
122	Inferior vena cava distensibility index (%)_T2
123	Inferior vena cava distensibility index (%)_T3
124	AHF_T1
125	AHF_T2
126	AHF_T3
127	Systolic blood pressure (mmHg)_T1
128	Systolic blood pressure (mmHg)_T2
129	Systolic blood pressure (mmHg)_T3
130	Diastolic Blood Pressure (mmHg)_T1
131	Diastolic Blood Pressure (mmHg)_T2
132	Diastolic Blood Pressure (mmHg)_T3
133	Mean arterial pressure (mmHg)_T1
134	Mean arterial pressure (mmHg)_T2
135	Mean arterial pressure (mmHg)_T3
136	Heart rate (bpm)_T1.1
137	Heart rate (bpm)_T2.1
138	Heart rate (bpm)_T3.1
139	Respiratory rate (rpm)_T1
140	Respiratory rate (rpm)_T2
141	Respiratory rate (rpm)_T3
142	Glasgow Coma Scale_T1
143	Glasgow Coma Scale_T2
144	Glasgow Coma Scale_T3
145	Sedation Scale (SAS)_T1
146	Sedation Scale (SAS)_T2
147	Sedation Scale (SAS)_T3
148	Temperature (°C)_T1
149	Temperature (°C)_T2
150	Temperature (°C)_T3

151	Anuria and/or RRT_T1
152	Anuria and/or RRT_T2
153	Anuria and/or RRT_T3
154	Na Ur_T1
155	Na Ur_T2
156	Na Ur_T3
157	K Ur_T1
158	K Ur_T2
159	K Ur_T3
160	Creat Ur_T1
161	Creat Ur_T2
162	Creat Ur_T3
163	Urine Output (mL/day)_T1
164	Urine Output (mL/day)_T2
165	Urine Output (mL/day)_T3
166	Is the patient under inotropic drugs_T1
167	Is the patient under inotropic drugs_T2
168	Is the patient under inotropic drugs_T3
169	* Norepinephrine (μg/kg/min)_T1
170	* Norepinephrine (μg/kg/min)_T2
171	* Norepinephrine (μg/kg/min)_T3
172	* Dobutamine (μg/kg/min)_T1
173	* Dobutamine (μg/kg/min)_T2
174	* Dobutamine (μg/kg/min)_T3
175	Is the patient under sedation drugs_T1
176	Is the patient under sedation drugs_T2
177	Is the patient under sedation drugs_T3
178	Midazolam (μg/kg/min)_T1
179	Midazolam (μg/kg/min)_T2
180	Midazolam (μg/kg/min)_T3
181	Propofol (μg/kg/min)_T1
182	Propofol (μg/kg/min)_T2
183	Propofol (μg/kg/min)_T3
184	Is the patient under other drugs_T1
185	Is the patient under other drugs_T2
186	Is the patient under other drugs_T3
187	Fluid Balance (ml)_T1
188	Fluid Balance (ml)_T2
189	Fluid Balance (ml)_T3
190	Tracheal Intubation_T1
191	Tracheal Intubation_T2
192	Tracheal Intubation_T3
193	Mode_T1
194	Mode_T2
195	Mode_T3
196	PEEP_T1
197	PEEP_T2
198	PEEP_T3
199	Pplat_T1
200	Pplat_T2

201	Pplat_T3
202	Respiratory rate_T1
203	Respiratory rate_T2
204	Respiratory rate_T3
205	Tidal volume (VT)_T1
206	Tidal volume (VT)_T2
207	Tidal volume (VT)_T3
208	Renal Replacement Therapy_T1
209	Renal Replacement Therapy_T2
210	Renal Replacement Therapy_T3
211	Hematocrit (%)_T1
212	Hematocrit (%)_T2
213	Hematocrit (%)_T3
214	Leukocytes (total*1000/mm^3)_T1
215	Leukocytes (total*1000/mm^3)_T2
216	Leukocytes (total*1000/mm^3)_T3
217	Creatinine (mg/dL)_T1
218	Creatinine (mg/dL)_T2
219	Creatinine (mg/dL)_T3
220	Acute Kidney Injury_T1
221	Acute Kidney Injury_T2
222	Acute Kidney Injury_T3
223	Na (mmol/L)_T1
224	Na (mmol/L)_T2
225	Na (mmol/L)_T3
226	K (mmol/L)_T1
227	K (mmol/L)_T2
228	K (mmol/L)_T3
229	Platelets (10^3/mm^3)_T1
230	Platelets (10^3/mm^3)_T2
231	Platelets (10^3/mm^3)_T3
232	Bilirubin (mg/dL)_T1
233	Bilirubin (mg/dL)_T2
234	Bilirubin (mg/dL)_T3
235	Glycemia (mg/dL)_T1
236	Glycemia (mg/dL)_T2
237	Glycemia (mg/dL)_T3
238	Prothrombin time (INR)_T1
239	Prothrombin time (INR)_T2
240	Prothrombin time (INR)_T3
241	Chloride_T1
242	Chloride_T2
243	Chloride_T3
244	aPTT_T1
245	aPTT_T2
246	aPTT_T3
247	PT_T1
248	PT_T2
249	PT_T3
250	Fibrinogen_T1 (g/L)

251	Fibrinogen_T2 (g/L)
252	Fibrinogen_T3 (g/L)
253	C-Reactive Protein_T1
254	C-Reactive Protein_T2
255	C-Reactive Protein_T3
256	CRP_Value (mg/L)_T1
257	CRP_Value (mg/L)_T2
258	CRP_Value (mg/L)_T3
259	PCT_Value (mg/mL)_T1
260	PCT_Value (mg/mL)_T2
261	PCT_Value (mg/mL)_T3
262	pH_T1
263	pH_T2
264	pH_T3
265	PaO2 (mmHg)_T1
266	PaO2 (mmHg)_T2
267	PaO2 (mmHg)_T3
268	PaCO2 (mmHg)_T1
269	PaCO2 (mmHg)_T2
270	PaCO2 (mmHg)_T3
271	HCO3 (mmol/L)_T1
272	HCO3 (mmol/L)_T2
273	HCO3 (mmol/L)_T3
274	Base Excess (mmol/L)_T1
275	Base Excess (mmol/L)_T2
276	Base Excess (mmol/L)_T3
277	FiO2_T1
278	FiO2_T2
279	FiO2_T3
280	PaO2/FiO2_T1
281	PaO2/FiO2_T2
282	PaO2/FiO2_T3
283	Sat O2 (%)_T1
284	Sat O2 (%)_T2
285	Sat O2 (%)_T3
286	Sat O2/FiO2_T1
287	Sat O2/FiO2_T2
288	Sat O2/FiO2_T3
289	Blood Cultures
290	Result
291	Sample 1
292	Microorganisms
293	Site of sampling
294	Sample 2
295	Microorganisms.1
296	Site of sampling.1
297	Sample 3
298	Microorganisms.2
299	Site of sampling.2
300	The patient was already receiving antibiotic treatment and this coverage has been maintained until the onset of sepsis

301	Result in ICU
302	Total Days in ICU
303	Hospital Result
304	Total Days in Hospital
305	SOFA_T1
306	SOFA_T2
307	SOFA_T3
308	APACHE II_T1
309	APACHE II_T2
310	APACHE II_T3
311	Troponine_T1
312	Troponine_T2
313	Troponine_T3
314	BNP_T1
315	BNP_T2
316	WBC abs count_T1
317	WBC abs count_T2
318	WBC abs count_T3
319	Lympho abs count_T1
320	Lympho abs count_T2
321	Lympho abs count_T3
322	Neutro abs count_T1
323	Neutro abs count_T2
324	Neutro abs count_T3
325	RBC count_T1
326	RBC count_T2
327	RBC count_T3
328	Platelet count_T1
329	Platelet count_T2
330	Platelet count_T3
331	Death due to withdrawl of care
332	Mortality 28days
333	Mortality 100days

Table 16: Full list of the ShockOmics dataset attributes.
